# MicroRNA-218-5p accelerates malignant behaviors of breast cancer through LRIG1

**DOI:** 10.1016/j.clinsp.2023.100302

**Published:** 2023-10-29

**Authors:** Juhang Chu, Luyao Huang, Yaru Wang, Lin Fang, Mingping Qian

**Affiliations:** a School of Medicine, Tongji University, China; b Department of Thyroid and Breast Surgery, Shanghai Tenth People's Hospital, School of Medicine, Tongji University, China

**Keywords:** Breast cancer, MiRNA-218-5p, LRIG1, Growth, Metastasis

## Abstract

**Objective:**

MicroRNAs play crucial roles in the pathogenesis of cancers. MiRNA-218-5p may act as either an oncogene or a tumor suppressor, but its role in the pathogenesis of Breast Cancer (BC) remains unclear.

**Methods:**

Infiltrative breast ductal carcinoma as well as corresponding adjacent normal samples were collected from 30 patients. Mimics and inhibitors of miRNA-218-5p or corresponding negative controls were transfected into BC cells. miRNA-218-5p expression was detected by quantitative PCR. The effects of miRNA-218-5p on the malignant behaviors of BC were assessed. Dual-luciferase reporter assay was employed to evaluate the binding of miRNA-218-5p to LRIG1.

**Results:**

BC tissues showed higher miRNA-218-5p expression as compared to the adjacent normal tissues. Ectopic miRNA-218-5p expression accelerated the cell cycle, cell growth and migration of BC, while repressed cell apoptosis. Interestingly, ectopic miRNA-218-5p expression down-regulated LRIG1 expression, and miRNA-218-5p could bind to LRIG1. Also, our study indicated that miRNA-218-5p up-regulated ErbB2 and EGFR expression by targeting LRIG1, suggesting that the LRIG1-mediated signaling pathway contributed to the pro-tumor effects of miRNA-218-5p on BC.

**Conclusion:**

MiRNA-218-5p up-regulates ErbB2 and EGFR expression by suppressing LRIG1 expression, thus promoting the malignant behaviors of BC. miRNA-218-5p may exert a pro-tumor effect on BC and serve as a therapeutic target for BC treatment.

## Highlights

• MiRNA-218-5p up-regulates ErbB2 and EGFR expression by suppressing LRIG1 expression.
• MiRNA-218-5p promotes the malignant behaviors of BC.
• MiRNA-218-5p may exert a pro-tumor effect on BC and serve as a therapeutic target for BC treatment.

## Introduction

MicroRNAs, a group of non-coding RNAs ranging 19‒24 nucleotides, are capable of controlling the expression of targeted genes by inducing degradation or inhibiting the translation of mRNA. In recent years, plenty of evidence has indicated that microRNAs are implicated in a variety of cellular processes, such as proliferation, death, and metastasis 1, 2, 3. Moreover, microRNAs may also exert anti-tumor or protumor effects, depending on their expressing patterns and downstream targets involved [4,5]. Some studies have revealed that miRNA-218-5p may act as an anticancer gene in various cancers, including renal cell carcinoma, hepatocellular carcinoma, gastric, colorectal and bladder cancers 6, 7, 8, 9, 10. However, the exact underlying mechanism by which miRNA-218-5p orchestrates the pathogenesis of Breast Cancer (BC) remains unclear.

Leucine-Rich-Repeats and Immunoglobulin Like domains 1 (LRIG1) is widely expressed in many healthy tissues [11], and its expression is down-regulated in a number of carcinomas such as BC, renal carcinoma and cervical cancer 12, 13, 14. LRIG is an inhibitor of some oncogenic receptor tyrosine kinases, including ErbB family [14] as well as the Met [15] and Ret receptor [16] members. The physiological significance of LRIG1 has been underscored in LRIG1 knock-out mice which showed up-regulated expression of ErbB and Met receptor 17, 18, 19.

This study aimed to investigate the role of miRNA-218-5p in the malignant behaviors of BC. Our results showed that miRNA-218-5p expression was significantly elevated in BC tissues. miRNA-218-5p over-expression accelerated cell growth and metastasis of BC, disrupted cell cycle, and suppressed cell apoptosis by targeting LRIG1. These findings indicate that miRNA-218-5p may confer a pro-tumor effect in BC.

## Methods

### Collection of human BC tissues

Pathologically confirmed infiltrative breast ductal carcinoma tissues were collected from 30 patients from the Department of General Surgery of the Shanghai Tenth People's Hospital (Shanghai, China) and corresponding adjacent normal tissues were obtained as controls. Patients did not receive chemotherapy or radiotherapy before surgery. This study was approved by the Ethics Committee of Shanghai Tenth People's Hospital (20KT156) and informed consent was obtained before the study. Reporting guidelines are not applicable, because it is not a clinical study.

### Cells and transfection

Human BC cell lines (MDA-MB-231, HCC1937, MCF-7, and MDA-MB-468) were from the American Type Culture Collection. The Chinese Academy of Science (Shanghai, China) kindly provided MCF-10A cells. Cells were grown in Dulbecco's modified Eagle's medium (DMEM; Gibco, USA) containing fetal bovine serum (FBS; 10%, Gibco), penicillin (100 U/mL), and streptomycin (100 μg/mL, Enpromise, China) at 37°C with 5% CO_2_.

For transfection, cells in serum-free medium were seeded into a 6-well plate (2 × 10^5^ per well). When cell confluence reached 30%–40%, 1 μg of miRNA-218-5p mimics/inhibitor/corresponding negative control (GenePharma Co., Ltd., China) were independently transfected into cells in the presence of lipofectamine transfection reagent (Invitrogen, USA). Cells were maintained for an additional 48 hours prior to further examination.

### RNA extraction and RT-qPCR

A miRNA quick extraction kit (Tiangen, Beijing, China) was used to extract miRNA from BC tissues and adjacent normal tissues. Trizol reagent (Invitrogen, USA) was employed for isolation of total RNA RNA was reverse transcribed via a reverse transcription PCR kit (TaKaRa, Japan). A relative quantity of RNA was detected with a quantitative PCR (RT-qPCR) kit (TaKaRa, Japan). GAPDH was used as an endogenous reference.

### Western blotting

Whole-cell proteins were extracted with protein lysis buffer (Sigma-Aldrich, USA) and quantified via a bicinchoninic acid assay (Pierce, USA). The proteins were separated through 10% sodium dodecyl sulfate-polyacrylamide gel electrophoresis and then transferred onto polyvinylidene difluoride membrane (EMD Millipore, USA). After blocking in non-fat milk, the membranes were incubated with antibodies against LRIG1-, ErbB2- or EGFR and then with horseradish peroxidase-conjugated secondary antibodies. Subsequently, an enhanced chemiluminescence detection kit (Millipore, USA) was employed for visualization. A Bio-RAD scanning system was applied to analyze the immunoreactive protein bands.

### Proliferation assay

Cell-counting kit 8 (Invitrogen, Shanghai, China) was used to detect cell proliferation according to the manufacturer's instructions. Approximately 4–5h after transfection with miRNA-218-5p inhibitor, miRNA-218-5p mimics, or negative control, cells were grown in 96-well plates (3000 cells/well) in triplicate. Cell growth was detected at 0h, 24h, 48h, and 72h. Then, the Optical Density (OD) was detected at 450 nm through a microplate spectrophotometer.

### Migration assay

Cell migration was assessed via transwell assay (Corning, USA). In brief, cells were suspended in serum-free DMEM (180 μL) and then added into the upper chambers (6 × 10^4^ per well). DMEM containing FBS (10%, 600 μL) was added to the lower chambers. 16h later, 3% paraformaldehyde was added to fix cells on the polycarbonate membranes, and cells were stained with 0.1% crystal violet for 15 min. A cotton swab was used to collect cells in the upper chambers and migrate cells adherent to the base of the chambers. Images were captured under an inverted microscope (Thermo Fisher Scientific, USA) at 200 × and five fields were randomly selected for further analysis.

### Flow cytometry

For cell cycle examination, after transfection with miRNA-218-5p inhibitor, miRNA-218-5p mimic or negative control, cells were collected and rinsed. Subsequently, ice-cold ethanol (3 mL) was added to cells, followed by incubation for over 30 min. After the addition of propidium iodide (PI; 0.05 g/L), cells were incubated at room temperature for 30 min, and then subjected to flow cytometry (Beckman coulter, USA).

Annexin V-FITC/PI cell apoptosis detection kit (BestBio, China) was employed for the detection of cell apoptosis. After transfection for 48h, cells were harvested, rinsed in PBS, and stained via Annexin V-FITC and PI. The percentages of apoptotic cells were determined by flow cytometer and analyzed using FlowJo software.

### Immunohistochemistry

Paraffin-embedded tissues were sectioned (4 μm), deparaffinized and re-hydrated. Endogenous oxidise activity was deactivated with 3% hydrogen peroxidase. After blocking, sections were incubated with primary antibodies for 1h at 4°C, and then with secondary antibodies for 30 min at room temperature. The sections were visualized with diaminobenzidine chromogen (Dako, Inc, US). Immunoreactivity (membrane-positive staining) was scored as follows: 0, 1+ (negative), 2+ (equivocal), and 3+ (positive).

### Dual-luciferase reporter assay

The 3′-UTR fragments of LRIG1 with predicted binding sites for miRNA-218-5p were cloned via PCR with PrimerSTAR Max DNA polymerase (Takara, Japan) and following primers: F:5′-GCGGAGCTCAACCAGAAGGCCAAGTC-3’, R:5′-GCGTCTAGAAAATGGACAAAGTGGGTGTGG-3′. LRIG1 3′-UTR was then inserted in the pmirGLO vector (Promega, USA) between XbaI and SacI. MCF-7 cells were seeded in a 24-well plate and grown with 1 μg of LRIG1 3′UTR reporter plasmid plus 30 nm of miRNA-218-5p mimics or negative control. Then, cells were lysed with PLB at 48h after incubation. The dual-luciferase reporter assay kit (Promega, USA) was applied for the detection of renilla or firefly luciferase signals.

### Statistical analysis

GraphPad Prism 5.0 software (GraphPad Software Inc., USA) was used for statistical analysis. Comparisons between two groups were performed with Student's *t*-test, whereas comparisons among groups were done with one-way analysis of variance. All experiments were carried out in triplicate, and data are expressed as mean ± Standard Deviation (SD). A value of p < 0.05 was considered statistically significant.

## Results

### MiRNA-218-5p expression in human breast cancer

**Figure 1:**
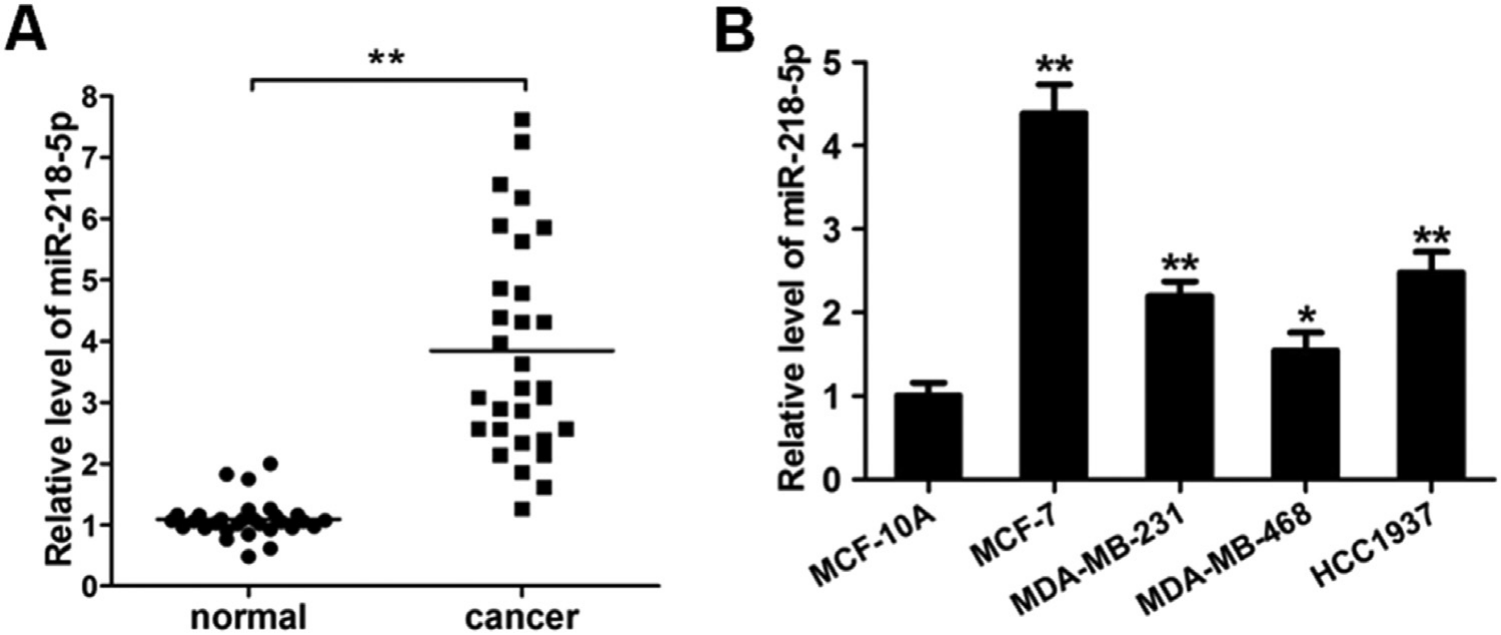
MiRNA-218-5p expression in BC tissues and cell lines. (A) MiRNA-218-5p expression in BC tissues and adjacent normal tissues (qRT-PCR). (B) MiRNA-218-5p expression in BC cell lines (MCF-7, MDA-MB-468, MDA-MB-231, HCC1937) and normal MCF-10A cells (qRT-PCR). Expression of miRNA-218-5p normalized to that of GAPDH (*p < 0.05, **p < 0.01).

The expression of miRNA-218-5p was detected in 30 BC tissues and corresponding adjacent normal tissues via RT-qPCR. As shown in Figure 1A, as compared to the adjacent normal tissues, the miRNA-218-5p expression was markedly increased in BC tissues. In HCC1937, MDA-MB-231, MCF-7, and MDA-MB-468 cells, miRNA-218-5p expression was also detected. Results showed the miRNA-218-5p expression in BC cell lines was markedly higher than in MCF-10A cells, an immortal mammary epithelial cell line (Fig. 1B), which was consistent with findings from *in vivo* investigation. MCF-7 and MDA-MB-231 cells were used in the following experiments.

### MiRNA-218-5p promotes malignant behaviors of BC cells

**Figure 2:**
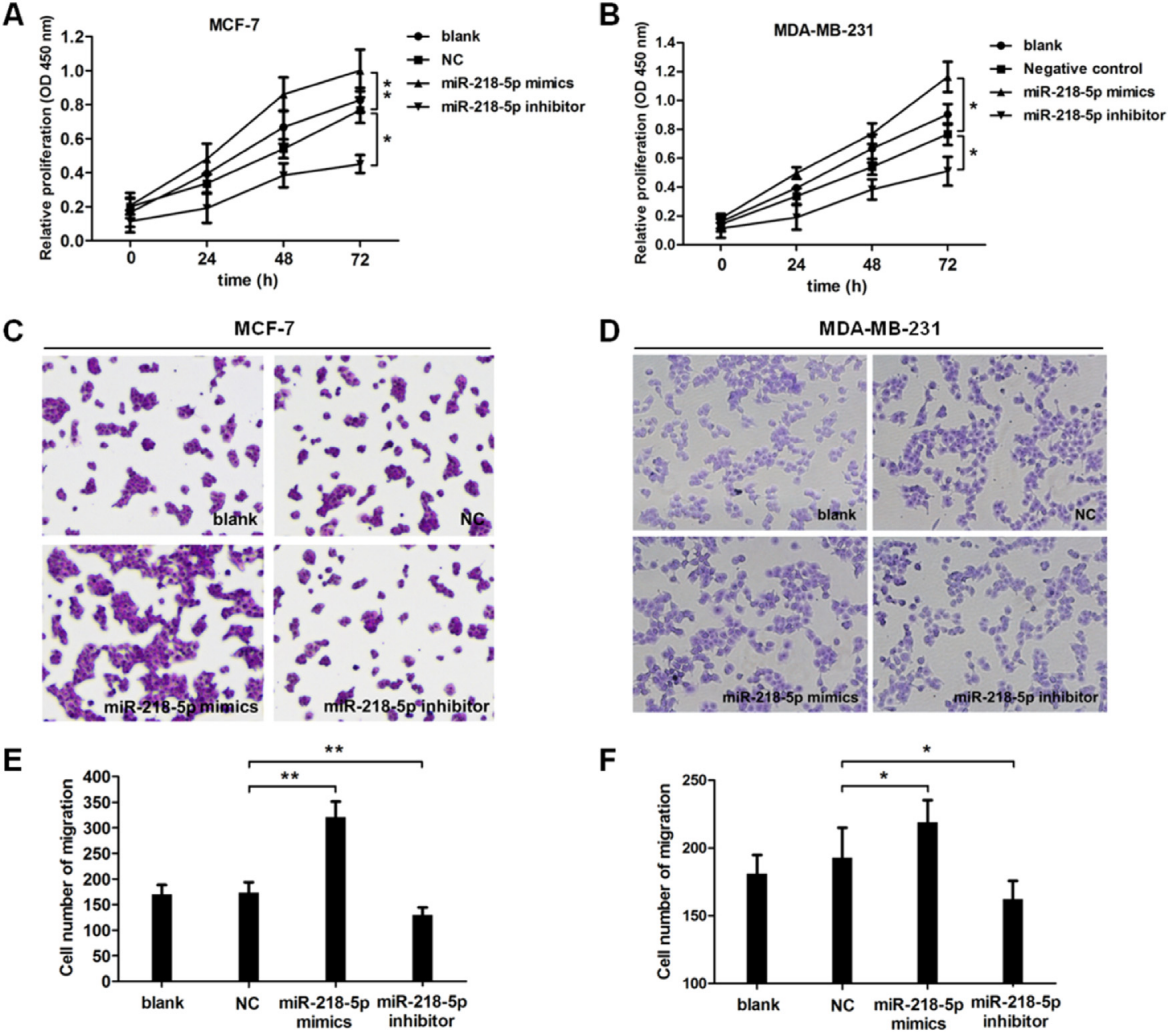
MiRNA-218-5p promotes growth of BC cells. Detection of cell growth (CCK8 assay) (A‒B). Examination of cell metastasis (Transwell assay) (C and E: MCF7; D and F: MDA-MB-231) (*p < 0.05, **p < 0.01). Data are expressed as means ± SEM.

To further investigate the effect of miRNA-218-5p on the malignant behaviors of BC, cell proliferation and migration were examined by CCK8 and Transwell assay, respectively. Results revealed that cell proliferation was accelerated upon miRNA-218-5p overexpression (Fig. 2A), while cell proliferation was suppressed upon miRNA-218-5p knockdown (Fig. 2B). In addition, the migration of BC cells was accelerated after miRNA-218-5p mimics transfection, while it was inhibited following miRNA-218-5p inhibitor transfection (Fig. 2 C‒F). In summary, miRNA-218-5p exerted proliferation-promoting and migration-promoting effects on BC cells.

### MiRNA-218-5p disrupts the cell-cycle progression of BC cells in different phases

**Figure 3:**
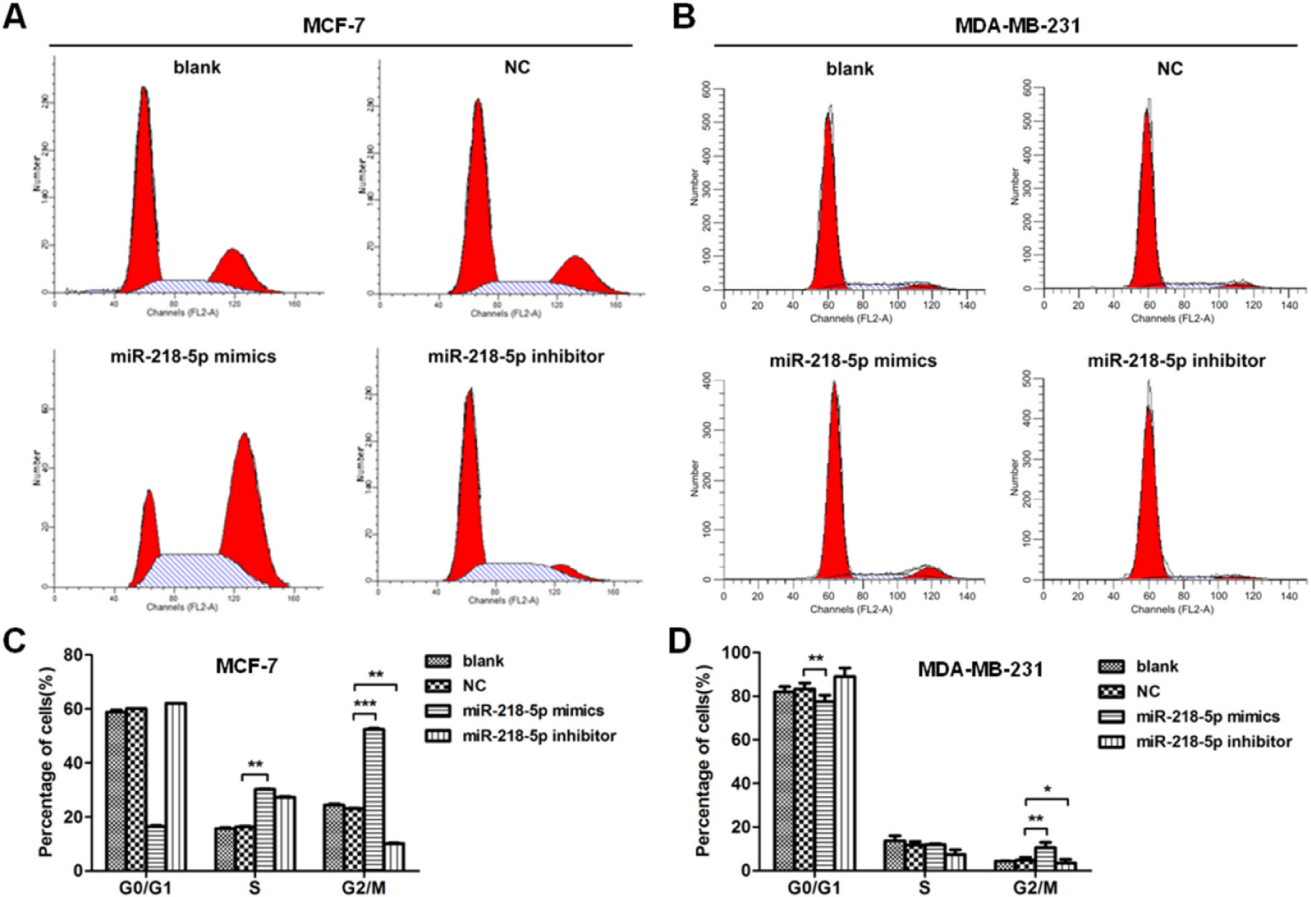
MiRNA-218-5p arrests BC cell cycle in S- and G2-phases. (A‒B) Detection of cell cycle after miRNA-218-5p mimics or miRNA-218-5p inhibitor transfecting for 36h (flow cytometry). (C‒D) Percentages of cells in G1-, S- and G2-phases (*p < 0.05, **p < 0.01).

The effect of miRNA-218-5p on the cell cycle progression was further investigated by flow cytometry. Results showed that miRNA-218-5p mimics transfection-arrested cell cycle in the G2/M-phase (Fig. 3 A‒D). On the other hand, attenuation of miRNA-218-5p significantly elevated the proportion of cells in the S-phase and reduced that in the G2/M-phase (Fig. 3 A‒D). These findings suggest miRNA-218-5p initiated S-phase arrest.

### MiRNA-218-5p inhibits BC cell apoptosis

**Figure 4:**
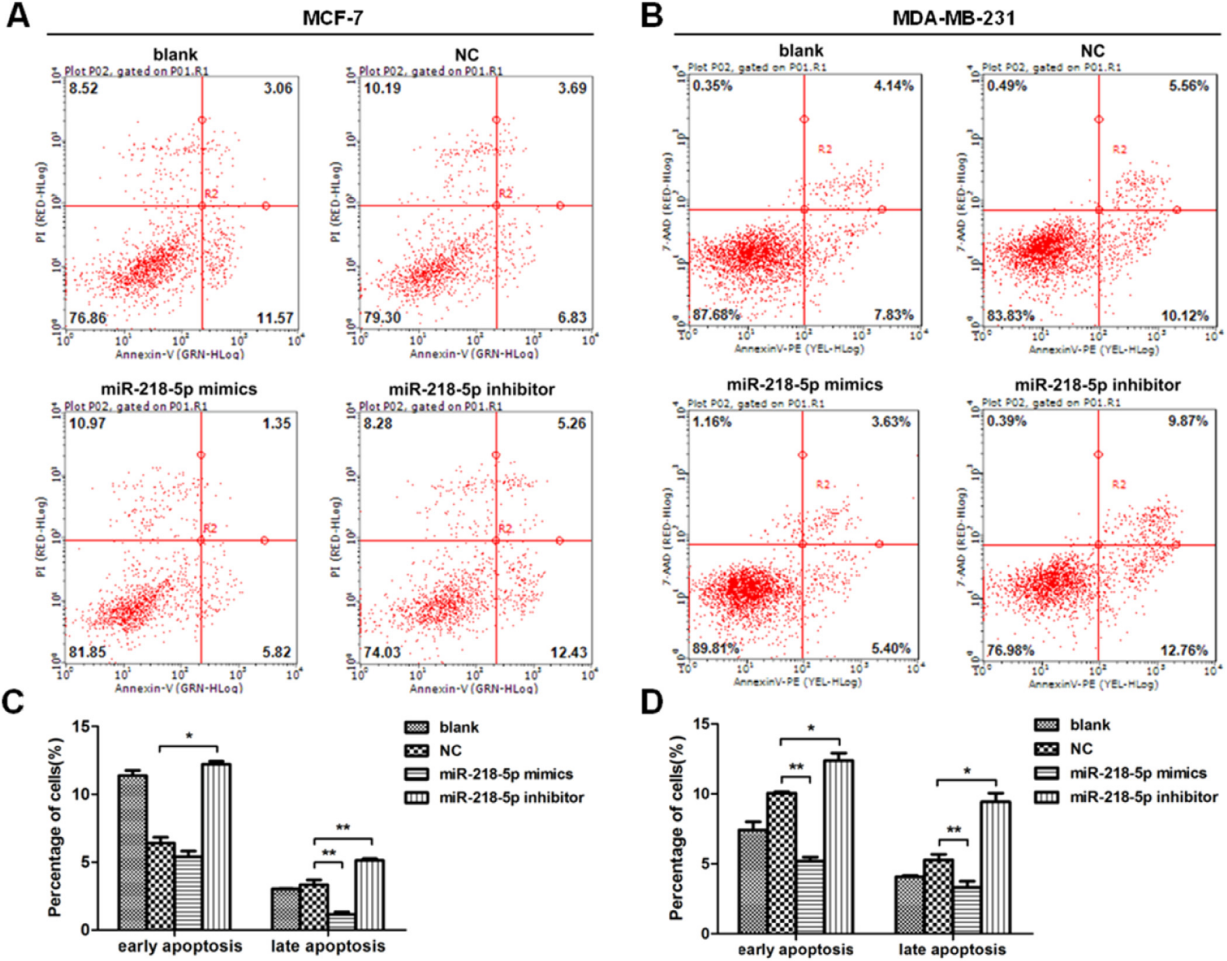
MiRNA-218-5p inhibits BC cell apoptosis at both early and late stages. (A‒B) Detection of apoptotic BC cells (flow cytometry). Proportion of cells in early and late apoptotic stages after miRNA-218-5p up-regulation (or down-regulation). (C: MCF7; D: MDA-MB-231) (*p < 0.05, **p < 0.01).

The apoptosis of BC cells after miRNA-218-5p up-regulation or down-regulation was further examined by flow cytometry. Results revealed that cells with miRNA-218-5p up-regulation showed a significantly lower proportion of late apoptotic cells as compared to the negative control (Fig. 4 A‒D). On the contrary, miRNA-218-5p down-regulation increased apoptotic cells, both in early and late apoptosis (Fig. 4 A‒D). In summary, these indicate that miRNA-218-5p can inhibit BC cell apoptosis.

### MiRNA-218-5p directly targets LRIG1

**Figure 5:**
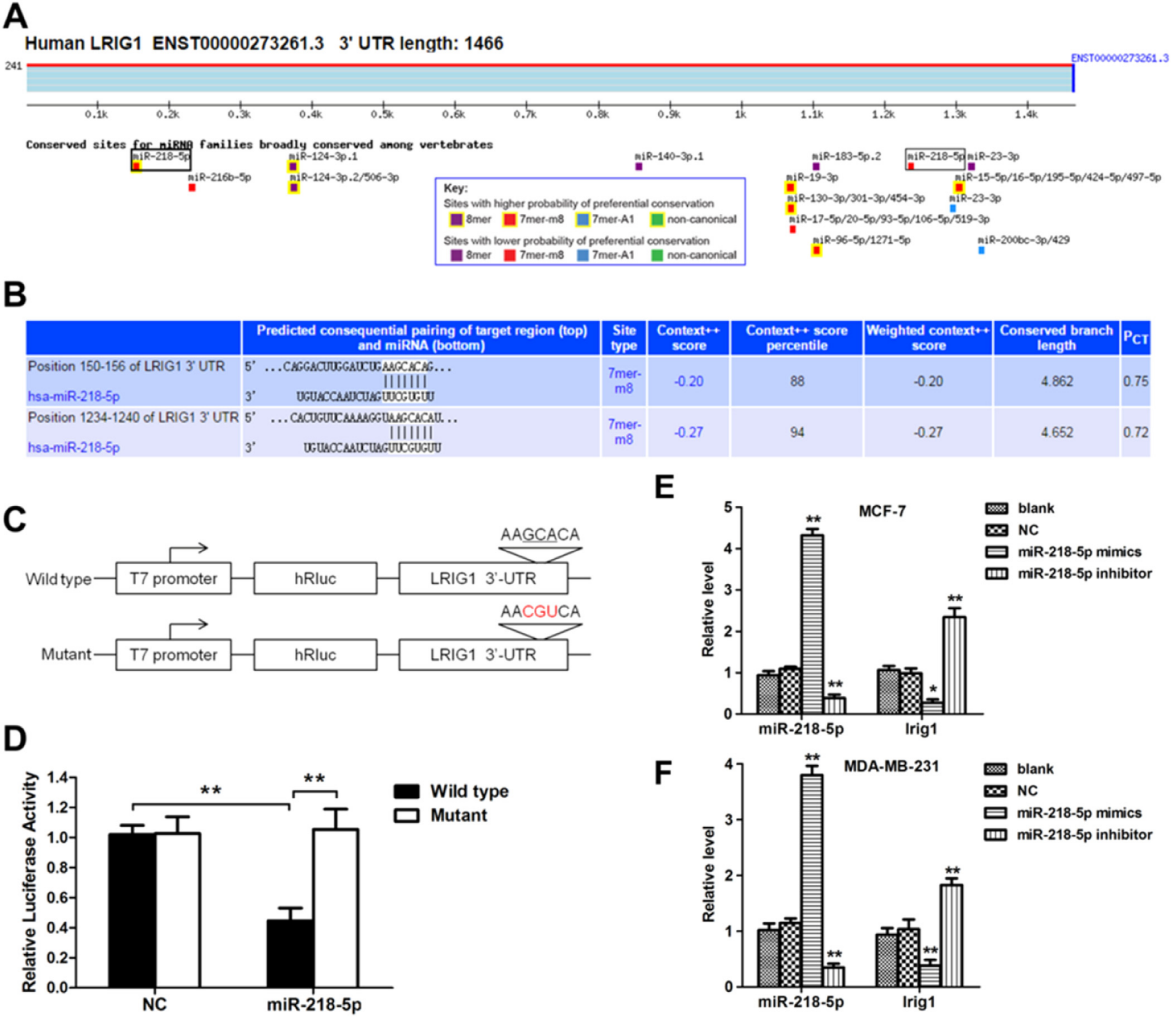
LRIG1 is a direct target of miRNA-218-5p. (A‒B) Bioinformatics analysis showed the potential target sites for miRNA-218-5p in LRIG1 3′-UTR. (C) Luciferase reporter plasmids containing miRNA-218-5p binding sites at LRIG1 3′-UTR were constructed. (D) After co-transfection with LRIG1 WT-UTR, LRIG1 mut-UTR plus miRNA-218-5p mimics or NC, the luciferase activity was analyzed. (E‒F) Forty-eight hours after transfection, the expression of miRNA-218-5p and LRIG1 was detected (* p < 0.05, **p < 0.01).

In order to investigate the mechanism by which miRNA-218-5p inhibited cell apoptosis, the influence of miRNA-218-5p on the LRIG1 expression was examined. MiRNA-218-5p transfection declined the LRIG1 mRNA expression, whereas miRNA-218-5p inhibitor transfection increased LRIG1 mRNA expression (Fig. 5 E‒F). These results inhibit the suppressive effect of miRNA-218-5p on the LRIG1 expression. Interestingly, prediction with TargetScan Release 6.2 software showed two miRNA-218-5p binding sites in LRIG1 3′-UTR (Fig. 5A), indicating that LRIG1 may be a potential target of miRNA-218-5p. The binding sites in Homo sapiens LRIG1 3′-UTR are shown in Fig. 5B. Then, luciferase reporter plasmids with the Wild-Type 3’-UTR (WT-UTR) or mutant miRNA-218-5p binding sites (mut-UTR) were constructed, aiming to investigate the binding of miRNA-218-5p to LRIG1 (Fig. 5C). Results showed miR-218-5p declined the luciferase activity of WT-UTR by up to 60%, without influence on mut-UTR luciferase activity (Fig. 5D). These findings indicate miRNA-218-5p suppresses LRIG1 expression via directly targeting LRIG1.

### ERBB2 and EGFR in LRIG1-mediated signaling pathway are downstream effectors of miRNA-218-5p

**Figure 6:**
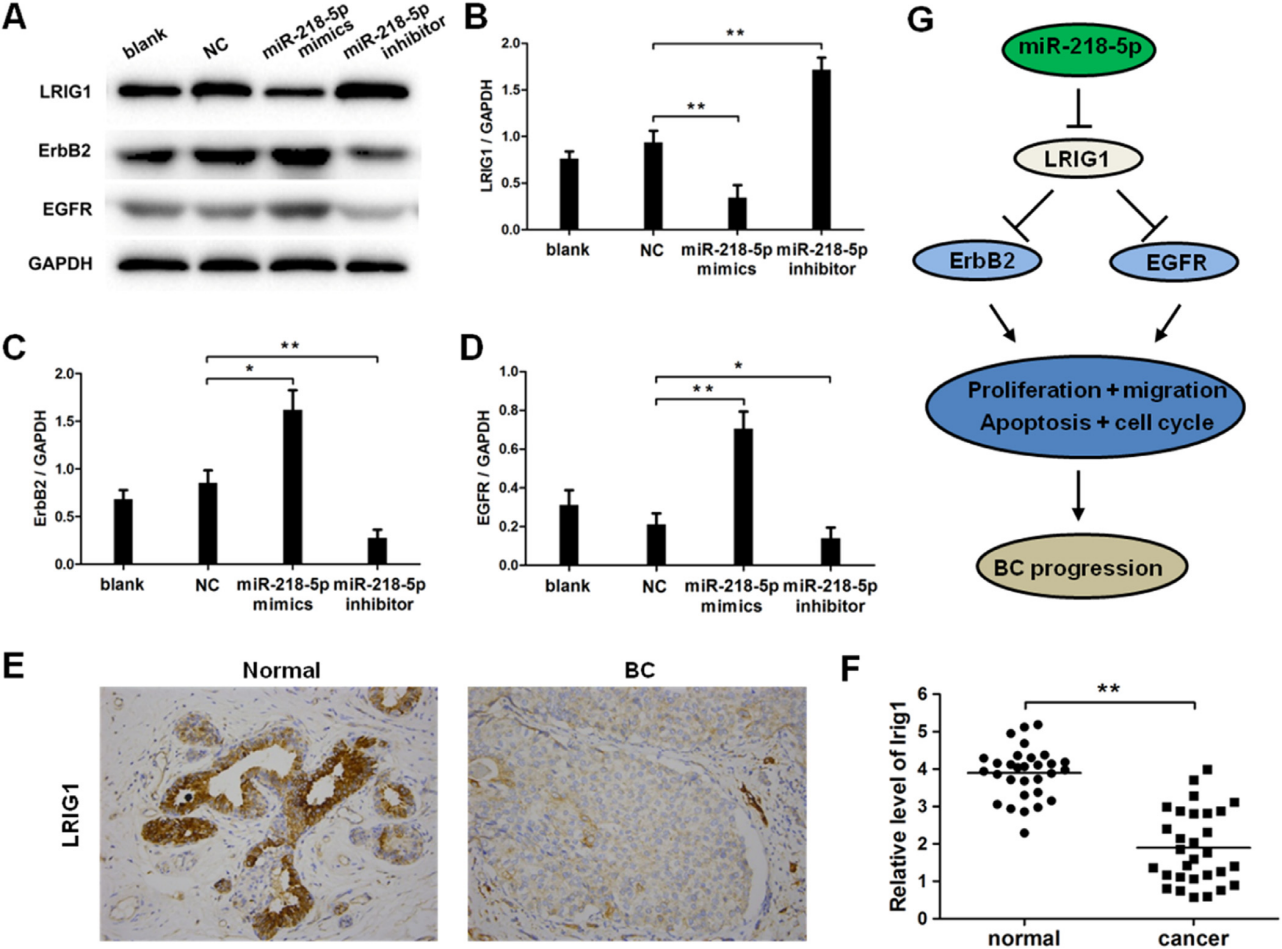
MiRNA-218-5p enhanced ErbB2 and EGFR expression in BC cells via suppressing LRIG1. The expression of LRIG1, ErbB2 and EGFR in MCF-7 cells with miRNA-218-5p up-regulation or down-regulation (Western blotting). (A) Representative images of Western blotting; (B: LRIG1; C: ErbB2, D: EGFR): Quantitative analysis of protein expression. (E) LRIG1 expression in BC tissues (immunohistochemistry). (F) LRIG1 expression in BC tissues and corresponding adjacent normal tissues (qRT-PCR). (G) Model of miRNA-218-5p function in BC cells (*p < 0.05, **p < 0.01).

EGFR and ERBB2 are downstream effectors in LRIG1 signaling, and therefore the expression of LRIG1, ERBB2, and EGFR was further detected after miRNA-218-5p up-regulation or down-regulation. Results indicated that miRNA-218-5p over-expression inhibited the mRNA expression of LRIG1 (Fig. 6 A‒B), and this suppressive effect was further confirmed by Western blotting and immunocytochemistry (Fig. 6 E‒F). Furthermore, miRNA-218-5p was proven to elevate the protein expression of ERBB2 and EGFR (Fig. 6 A, C, and D). As LRIG1 is a target of miRNA-218-5p, our results indicate ERBB2 and EGFR are downstream effectors of miRNA-218-5p, at least partially induced by targeting LRIG1 (Fig. 6G).

## Discussion

Studies have reported that miRNAs can exert anti-tumor or pro-tumor effects, and the expression of some miRNAs is altered during the development of tumors. 20, 21, 22, 23 The dysfunction of miRNA in various cancers indicates that modulating miRNA expression may become a new therapeutic treatment for cancers. Up to now, miRNA-targeting therapy has been employed by using miRNA sponges, antisense oligonucleotides, or small-molecule inhibitors. Chemically synthesized miRNAs or oligonucleotides targeting miRNAs have been proven to efficiently inhibit cancer development. 24, 25, 26, 27 At this time, several preclinical and clinical trials on miRNA-targeting therapy shedding light on cancer treatment are ongoing. 28, 29, 30

MiRNAs are vital for the tumor development of BC. 31, 32, 33 This study investigated the role of miRNA-218-5p in the pathogenesis of BC. Our results showed miRNA-218-5p expression in human BC tissues was markedly upregulated as compared to adjacent normal tissues. Similar findings have been reported in other cancers, indicating that increased miRNA-218-5p expression may be a common event in human cancers. 34, 35, 36 In the subsequent experiment, mimics of miRNA-218-5p were transfected into MCF-7 cells, and results showed that cell growth was markedly accelerated and cell migration was significantly promoted. All these findings indicate that miRNA-218-5p promotes the growth and migration of BC cells. In addition, miRNA-218-5p inhibited the cell cycle of BC cells. However, no marked difference was noted in apoptotic cells between miRNA-218-5p and negative control.

To reveal the underlying mechanism by which miRNA-218-5p exerts effects on cell growth, potential targets were searched in the miRNA Base, target scan, and miRNA and a database. LRIG1 was predicted to be a direct target of miRNA-218-5p, which was finally confirmed through luciferase reporter assay. In addition, LRIG1 mRNA and protein expression was dramatically lower in the miRNA-218-5p overexpression group than in the negative control group. These findings indicate that LRIG1 is a downstream target of miRNA-218-5p.

Studies have reported that LRIG1 can regulate ErbB family RTKs on cell surfaces. [37] In tamoxifen-treated luminal BCs, up-regulation of LRIG1 suppresses RTK family expression and signaling, including EGFR and ErbB2-4. [38] Our findings indicated that miRNA-218-5p up-regulated ErbB2 and EGFR expression by targeting LRIG1, suggesting that the LRIG1-mediated signaling pathway contributed to the effects of miRNA-218-5p on the malignant behaviors of BC.

## Conclusions

Collectively, our study reveals that miRNA-218-5p may disrupt the cell cycle, induce cell growth and metastasis of BC cells via regulating LRIG1. These findings indicate that miRNA-218-5p may exert a pro-tumor effect on BC. Furthermore, LRIG1 is a downstream target of miRNA-218-5p, and therefore decreasing miRNA-218-5p or upregulating LRIG1 may serve as new treatments for BC.

## Authors' contributions

JC designed the work, collected, and analyzed data, and drafted the manuscript; LH, YW, and LF collected and analyzed data and revised the manuscript; and MQ contributed to the conception and design of the work, reviewed and revised the manuscript.

## Funding

This work was supported by the 10.13039/501100001809National Natural Science Foundation of China (no. 82073204). The authors thank all subjects in this study. The authors also thank all the doctors and nurses from the Department of Thyroid and Breast Surgery at Shanghai Tenth People's Hospital in China for their contributions.

## Declaration of Competing Interest

The authors declare no conflicts of interest.

## Data Availability

The datasets generated for this study are available on request to the corresponding author.
